# Feasibility of Solar Updraft Towers as Photocatalytic Reactors for Removal of Atmospheric Methane–The Role of Catalysts and Rate Limiting Steps

**DOI:** 10.3389/fchem.2021.745347

**Published:** 2021-09-10

**Authors:** Yanfang Huang, Yimin Shao, Yang Bai, Qingchun Yuan, Tingzhen Ming, Philip Davies, Xiaohua Lu, Renaud de Richter, Wei Li

**Affiliations:** ^1^ Department of Chemical and Biological Engineering, Nantong Vocational University, Nantong, China; ^2^ Institute for Materials and Processes, School of Engineering, The University of Edinburgh, Edinburgh, United Kingdom; ^3^ School of Engineering and Applied Science, Aston University, Birmingham, United Kingdom; ^4^ School of Civil Engineering and Architecture, Wuhan University of Technology, Wuhan, China; ^5^ School of Engineering, University of Birmingham, Birmingham, United Kingdom; ^6^ State Key Laboratory of Materials-Oriented Chemical Engineering, College of Chemical Engineering, Nanjing Tech University, Nanjing, China; ^7^ Tour-Solaire.fr, Montpellier, France

**Keywords:** solar updraft, photocatalysis, non-CO_2_ greenhouse gases, methane, climate change

## Abstract

Due to the alarming speed of global warming, greenhouse gas removal from atmosphere will be absolutely necessary in the coming decades. Methane is the second most harmful greenhouse gas in the atmosphere. There is an emerging technology proposed to incorporating photocatalysis with solar updraft Towers (SUT) to remove methane from the air at a planetary scale. In this study, we present a deep analysis by calculating the potential of methane removal in relation to the dimensions and configuration of SUT using different photocatalysts. The analysis shows that the methane removal rate increases with the SUT dimensions and can be enhanced by changing the configuration design. More importantly, the low methane removal rate on conventional TiO_2_ photocatalyst can be significantly improved to, for example, 42.5% on a more effective Ag-doped ZnO photocatalyst in a 200 MW SUT while the photocatalytic reaction is the rate limiting step. The factors that may further affect the removal of methane, such as more efficient photocatalysts, night operation and reaction zone are discussed as possible solutions to further improve the system.

## Introduction

One of the grand challenges humankind is facing is global warming. The 2018 report of Intergovernmental Panel on Climate Change (IPCC) emphasizes the need for “rapid and far-reaching” actions now to curb carbon emission to limit global warming and climate change impact (Masson-Delmotte et al., 2018). Countries all around the globe are setting up ambitious target to reach carbon neutrality.

Several anthropogenic emissions which are extremely difficult to eliminate (i.e. from aviation or from agriculture) will need to be balanced by negative emission technologies (NETs) to achieve overall neutrality.

On the one hand, the NETs proposed until today are mainly based on carbon dioxide removal (CDR), but require safe and reliable sequestration of billions of tons of CO_2_, including capture, purification, compression and transportation to the storage sites (Kuramochi et al., 2012; Leung et al., 2014; Wetenhall et al., 2014; Kolster et al., 2017).

On the other hand, few greenhouse gas (GHG) removal methods of the other GHGs (methane CH_4_, nitrous oxide N_2_O, and ozone layer depleting gases included in the Montreal protocol like hydrochlorofluorocarbons, hydrofluorocarbons and chlorofluorocarbons) have yet been proposed, but have the advantage that these GHGs can be transformed or destroyed into benign gases and consequently don’t require sequestration and storage (United States Environmental Protection Agency (US EPA), 2013; Sikkema and dissertation, 2013; Jackson et al., 2019).

Moreover, the Global Warming Potential (GWP) of many of the non-CO_2_-GHGs are much higher than that of CO_2_. For example, the GWP of methane is nearly 28 times higher than that of CO_2_ on a 100-years basis, and 84 times higher on a 20-years basis (Masson-Delmotte et al., 2018).

In the atmosphere, the main processes by which the majority of non-CO_2_-GHGs are destroyed or transformed into benign gases are 1) hydroxyl radical oxidation, 2) photolysis and 3) reaction with halogen atoms. Photocatalytic processes (reactions activated by photons and accelerated by catalysts), have been shown to be able to transform almost all GHGs into benign gases (de Richter and Caillol, 2011) and even to reduce and transform CO_2_ to fuels or useful chemicals (Brudvig and Campagna, 2017; Hisatomi and Domen, 2017).

Many efficient photocatalytic materials have been explored in recent years (Liu et al., 2019; Sekar et al., 2019; Aljaafari et al., 2020; Qi et al., 2020a; Qi et al., 2020b; Qi et al., 2020c; Qi et al., 2020d; Jiang et al., 2020; Wang et al., 2020; Zada et al., 2020; Li et al., 2021; Sekar et al., 2021; Zhang et al., 2021; Zhou et al., 2021). Several of them are very efficient for the total oxidation of methane, including several TiO_2_ modified derivatives and other metal oxides (Jin et al., 2017). Chen et al. demonstrated a cheap ZnO derivative doped with 0.1% silver that efficiently oxidize methane under ambient conditions with high stability in this particular gas phase application (Chen et al., 2016). Minami et al. studied the oxidation of methane by TiO_2_ (Minami and Kim, 2006). They concluded that, within the concentration range studied, the decomposition reactions were first-order, with activation energy of 16.6 kJ/mol for methane, derived from the overall reaction rate constant. Krishna et al. reported another photocatalyst (i.e. uranyl-anchored MCM-41) and showed high activity for the total oxidation of methane under sunlight at ambient conditions (Krishna et al., 2004). Foam-nickel coated by TiO_2_ can photocatalytically oxidize air pollutants and can be applied to various household systems for heating, ventilation and air-conditioning, designed by Yang et al. (2009). Some other researchers like Kleinschmidt, Haeger et al., provided more insights into the kinetic of the oxidation reactions from methane to fully oxidized CO_2_ (Kleinschmidt and Hesse, 2002; Haeger et al., 2004a; Haeger et al., 2004b).

**FIGURE 1:**
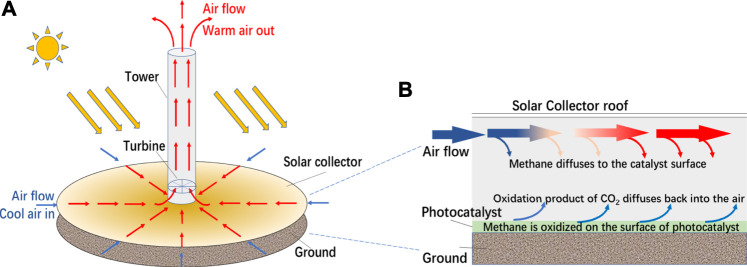
**(A)** The structure of SUT, **(B)** a conceptual diagram of the integrated system for methane removal through SUT.

The above photocatalysts for methane oxidation were only tested in very small lab scale. In order to reduce significantly the atmospheric concentration of CH_4_, it is necessary to process significant volumes of air at planetary scale. In a previous report (de Richter et al., 2017), the authors, for the first time, proposed to use solar updraft towers (SUTs) for this purpose, which consist of a large glazed solar greenhouse supplying warm air to a tall chimney where the airflow is induced by the stack effect (Haaf et al., 1983; Haaf, 1984; Cao et al., 2015; Liu et al., 2021; Ming et al., 2021). By coating the glazed canopy and the ground of the greenhouse by a photocatalyst, the SUT may be modified as a giant photocatalytic reactor, as shown in Figure 1.

Using the above lab scale data to analyze the performance of photocatalysis in giant SUTs is the essential first step to understand the feasibility of this proposed NET. A preliminary analysis based on heat transfer models indicated that it would remove 2 out of 3 CH_4_ molecules entering (de Richter et al., 2017). This was based on experimental data collected from the biggest SUT prototype ever built, by extrapolation of measured heat transfer data to estimate the potential effectiveness of mass transfer if the device is used as photocatalytic reactors.

A more comprehensive and reliable analysis is much needed. Besides mass transfer via the boundary layers between the bulk flow and the surface of the catalyst, several other factors would in practice limit the effectiveness of the process, local adsorption, surface reaction, desorption kinetics, quantum yield, etc.

By deeper analysis of the main relevant processes, this study, for the first time, presents such a comprehensive and reliable analysis of the effectiveness of SUT enabled photocatalysis for methane removal at planetary scale.

## Theory

**FIGURE 2:**
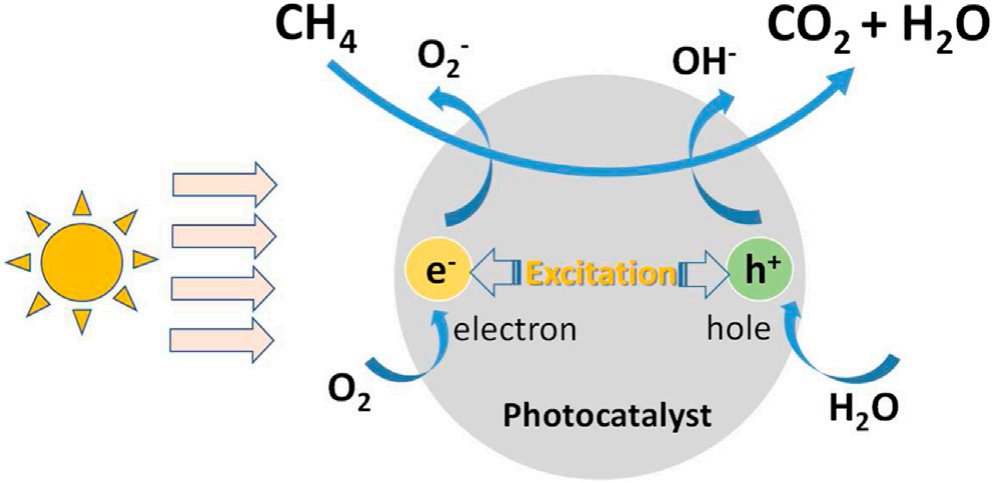
The mechanism diagram of photocatalytic oxidation of methane.

Photocatalytic degradation of atmospheric methane is a heterogeneous catalytic reaction, which happens at the interface between air and photocatalyst when methane flows through the surface of photocatalyst with air. The mechanism diagram of the reaction is shown in Figure 2 and the process can be generally divided into the following six steps:

1) Diffusion of methane to the surface of Photocatalyst;
2) Adsorption of methane on the surface of photocatalyst;
3) Hole-photon pair excited by light irradiation;
4) Methane molecules react on the surface of photocatalyst to produce products;
5) Desorption of products from the surface of photocatalyst;
6) Diffusion of products from the surface of photocatalyst to bulk gas.

There are both physical changes and chemical reactions in the above six steps, among which 1) and 6) are convection/diffusion processes, 2) and 5) are adsorption and desorption on the surface, 3) are photon excitation processes, and 4) are surface photocatalytic reaction. 2), 4) and 5) are regarded as three basic steps of heterogeneous catalysis. It is worth noting that the conversion of CO_2_ and H_2_O to CH_4_ is not observable in this process because it is largely an oxidation atmosphere rather than reduction atmosphere in ambient conditions.

In order to effectively apply SUT to methane mitigation in the air, an understanding of the relevant mass transfer and kinetic phenomena is required.

## Results and Discussion

### How Much CH_4_ Flow Through SUT?

The analyses are based on the experimental results of the SUT in Spain collected in 1982 (Haaf et al., 1983; Haaf, 1984). The key dimensions and data of the Spanish prototype are shown in Table 1.

**TABLE 1:** Key dimensions and data of the Spanish pilot plant.

Parameter	Value
Height of tower, H_T_	194.6 m
Radius of tower, R_T_	5.08 m
Mean radius of collector, R_c_	122 m
Average height of canopy, H_c_	1.85 m
Solar radiation	1000 W/m^2^
Ambient temperature	302 K
Temperature rise	20 K
Velocity of air flow (load conditions)	9 m/s
Velocity of air flow (release)	15 m/s
Power output	50 kW

There are two different places to coat photocatalyst, on the ground or on the surface under the canopy. In the following analysis, we assume that the photocatalyst is coated on the ground surface. Air is supplied to the chimney from the entrance of the collector similar to a greenhouse along the radial direction. In this process, it contacts the photocatalyst coated on the ground and the methane is degraded. Based on the data in Table 1, it is estimated that the amount of air flowing through the collector is 729 m^3^/s. If the concentration of methane in the atmosphere is 1.8 ppm, it means nearly 74.5 kg of methane per day and 761 Tonnes of CO_2_ equivalent per year flowing through this SUT.

Furthermore, if photocatalysis is combined with an up-scaled SUT of 200 MW (i.e. R_c_ = 3,500 m, H_T_ = 1,000 m and R_T_ = 65 m), it can provide 38 km^2^ GH area. According to Schlaich et al.’s report (Schlaich et al., 2005), despite the considerable dimensional differences between a 200 MW SUT and the Spanish 50 kW SUT, the main thermodynamic parameters are similar in both cases. Therefore, it is reasonable to assume that the 200 MW SUT has the same temperature rise and tower outlet velocity as the Spanish SUT. The amount of air passing through the collector can be calculated as 2 × 105 m^3^/s. The amount of methane is 2.0 × 104 kg per day, and nearly 2.1 × 10^5^ Tonnes of CO_2_ equivalent per year flowing through a single SUT.

### Kinetics of Methane Oxidation

In order to simplify the problem, there are two assumptions: 1) only methane reacts on the surface, and there is no multi-component competitive reaction; 2) the reaction is uniform along the radial direction at the same R (R is the distance from the collector center).

Many researchers have described the adsorption and photocatalytic reaction step for CH_4_ total oxidation. The following analysis is based on data form Haeger et al. (2004a) and Chen et al. (2016).

In Haeger et al.’s report (Haeger et al., 2004b), the kinetics of the total oxidation of methane were studied in a continuous-stirred tank reactor with titanium dioxide as the photocatalyst. The model established by Andreas Haeger et al. can be expressed as,

$$r_{AI}=B\frac{B_{1}C\left(CH_{4}\right)}{1+B_{1}C\left(CH_{4}\right)}\cdot \frac{B_{2}C\left(O_{2}\right)}{1+B_{2}C\left(O_{2}\right)}.$$ (1)

**FIGURE 3:**
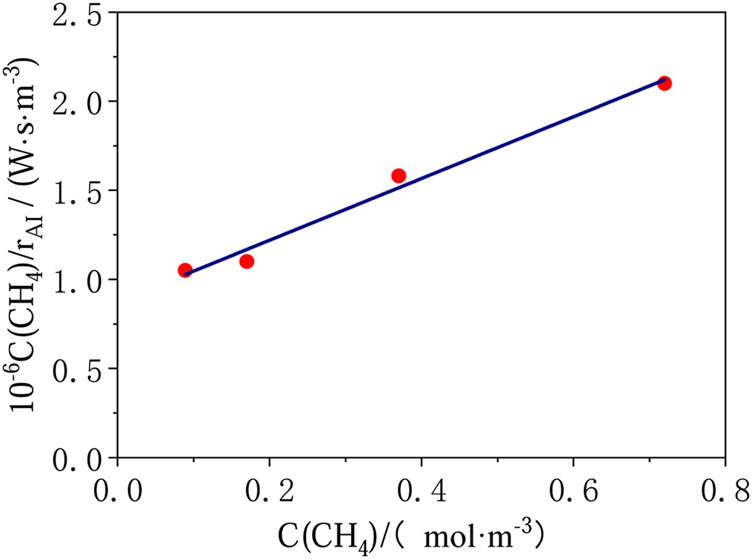
Results of photocatalytic total oxidation of methane for oxygen rich mixtures (T = 313 K, *p* = 1 bar, I = 14.5 W/m_2_, C(O_2_) = 6.81 mol/m^3^).

The parameter values in the above model are as follows:

$$\begin{array}{l} B=0.537\times 10^{-6}\text{ mol}\cdot {\text{W}}^{-1}{\text{s}}^{-1}\\ B_{1}=2.42\;{\text{m}}^{3}/\text{mol}\\ B_{2}=4.60\;{\text{m}}^{3}/\text{mol}\text{,} \end{array}$$ (2)

where, *r*_*AI*_ is the reaction rates per absorbed irradiation intensity and geometric surface area of the active plate, mol·W^−1^ s^−1^. The product of *r*_*A*I_ and light intensity I is the reaction rate r(CH_4_), mol·m^−2^ s^−1^; C(CH_4_)and C(O_2_) are the concentration of CH_4_ and O_2_ respectively, mol/m^3^. Plotting C(CH_4_)/*r*_*AI*_ vs. C(CH_4_), a straight line is achieved (Figure 3).

If the zenith solar intensity is 1120 W/m^2^, the intensity of light with wavelength less than 400 nm can be estimated to be 56 W/m^2^ according to the solar spectrum at sea level. Eq. 1 is used to predict initial reaction rate of 4.8 × 10^–9^ mol m^−2^ s^−1^ at atmospheric methane concentration of 1.8 ppm.

For the 50 kW SUT in Spain, the ground under collector provides 46735 m^2^ reaction area. It means that 2.2 × 10^–4^ mol/s of methane can be removed (i.e. 0.31 kg/day), which is far less than the amount of methane flowing through the collector at nearly 74.5 k/day (i.e. 0.4% of removal rate).

If photocatalysis is combined with 200 MW SUT, the methane removal capacity is 0.18 mol/s, that is, 252 kg/day of methane removal, which is also less than the amount of methane flowing through the collector at 2.0 × 104 kg/day (i.e. 1.3% of removal rate).

**FIGURE 4:**
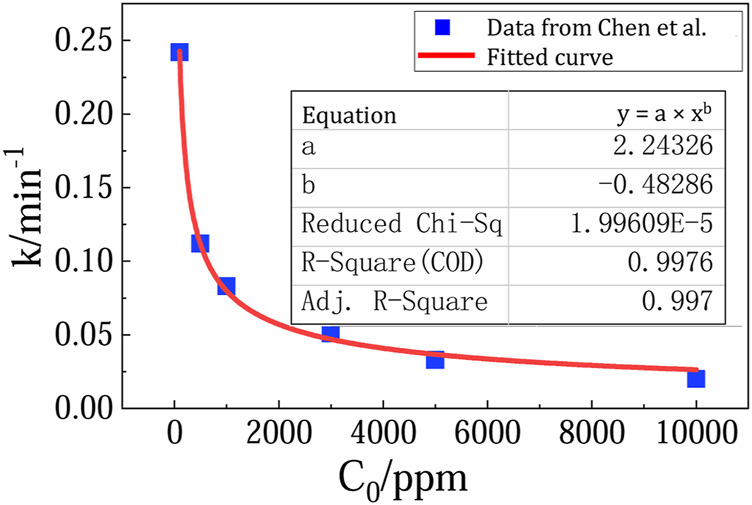
Fitting curve of C_0_ and k from experimental data.

Fortunately, beside the less effective TiO_2_ photocatalyst, there are recent progress on much more effective new photocatalysts for methane oxidation. For example, in Chen et al.’s report (Chen et al., 2016), the photocatalytic methane oxidation over ZnO doped with 0.1 wt% Ag (noted as 0.1-Ag-ZnO) follow pseudo-first-order kinetics. A fitting equation can be obtained by plotting k (the rate constant) with C_0_ (the initial methane concentration) (Figure 4). The rate constant k and the initial reaction rate can be predicted to be 1.69 min^−1^ and 3.04 ppm/min at atmospheric methane concentration of 1.8 ppm. Considering the area of the reaction surface (a 7 cm diameter disc) and the volume of reactor (a 0.45 L cylinder vessel), it is estimated that the reaction constant k and initial reaction rate are 0.0035 m/s and 2.36 × 10^–7^ mol m^−2^ s^−1^, respectively.

Calculations of methane removal rate based on 0.1-Ag-ZnO photocatalysts is summarized in Table 2 and compared with those based on TiO_2_. In the 50 kW SUT, methane can be removed at a rate of 15.2 kg/day (i.e. 20% of removal rate). And in a 200 MW SUT, methane can be removed at a rate of 1.24 × 104 kg/day (i.e. 62% of removal rate).

**TABLE 2:** Methane removal in different sizes of SUT using different photocatalysts.

Photocatalysts	SUT size	Removal rate kg/day	Removal rate %	Removal rate tonne CO_2_ e/year
TiO_2_	50 kW	0.31	0.4	
	200 MW	252	1.3	
0.1-Ag-ZnO	50 kW	15.2	20	
	200 MW	1.24 ×10^4^	62	1.27 ×10^5^

### Mass Transfer

Because of the concentration gradient of methane between bulk fluid and photocatalyst surface, there is a mass transfer flux which are controlled by advection and diffusion (Fogler, 2016). For the present case, the direction of air flow is radial and parallel to the ground, so the mass transfer caused by bulk fluid motion does not need to be considered. The molar diffusive flux of methane can be written as following equation:

$$W\left(CH_{4}\right)=h_{m}\left(C\left(CH_{4}\right)-C_{s}\left(CH_{4}\right)\right),$$ (3)

where C(CH_4_) is the averaged methane concentration, mol/m^3^; C_s_(CH_4_) is the methane concentration in the air next to the air–photocatalyst interface, mol/m^3^; h_m_ is the mass transfer coefficient, m/s.

Because heat transfer and mass transfer are analogous, correlations for Nusselt and Sherwood numbers are also analogous. Therefore, when the correlation for Nusselt number (Nu), Prandlt number (Pr) and heat transfer coefficient (α) is found based on the data from experiments, the correlation for Sherwood number (Sh), Schmidt number (Sc) and mass transfer coefficient (h_m_) can be obtained by simple replacement. If the Pr equals Sc, then Nu equals Sh.

For 50 kW SUT, based on the temperature rise, the heat transfer rate is 24,300 kW (de Richter et al., 2017), heat transfer coefficient can be calculated as below

$$\alpha =\frac{Q}{A\;\Delta T},$$ (4)

where *Q* is the heat transfer rate, kW; *A* is the heat transfer area, m^2^; and Δ*T* is the logarithmic mean temperature, K.

$$\Delta T=\frac{\left(T_{g}-T_{air,in}\right)-\left(T_{g}-T_{air,out}\right)}{\text{ln}\frac{T_{g}-T_{air,in}}{T_{g}-T_{air,out}}},$$ (5)

*T*_*g*_ is the temperature of the ground, *T*_*air,in*_ is the inlet temperature and *T*_*air,ou*t_ is the outlet temperature of the collector respectively. Nu number can be calculated by

$$Nu=\frac{\alpha L_{char}}{\lambda },$$ (6)

where *L*_*char*_ is the characteristic length, m, *L*_*char*_ = 2H_c_. *λ* is air thermal conductivity, W·m^−1^K^−1^. According to the definition of Sh, Eq. 7 can be used to calculate the mass transfer coefficient *h*_*m*_.

$$h_{m}=\frac{Sh\;D_{CH_{4},air}\;}{L_{char}},$$ (7)

where *D*_*CH*4, air_ is the diffusion coefficient, m^2^/s.

If the concentration of methane on the surface of photocatalyst is assumed to be zero and the concentration of methane in the bulk air is kept at 1.8 ppm, the average rate of mass transfer is 1.1 × 10^–6^ mol m^−2^ s^−1^ calculated from Eq. 3. The main parameters and calculation results are summarized in Table 3.

**TABLE 3:** The key parameters and calculation results.

Parameter or calculation results	Value
Density of air, *ρ*	1.1 kg/m^3^
Thermal conductivity of air, λ	0.024 W m^−1^K^−1^
Temperature of the ground	343 K (Haaf 1984)
Log mean temperature, ΔT	30 K
Heat transfer coefficient, α	17 W m^−2^K^−1^
Viscosity of air	17.9 × 10^–6^ Pa s
Diffusion coefficient, D_AB_	2.29 × 10^–5^ m^2^/s (Massman 1998)
Nu	2,620
Sh	2,620
Mass transfer coefficient, h_m_	0.016 m/s

Sherwood (Sh) number correlations can also be expressed by *Sh = constant × Re* ^*m*^ *× Sc* ^*n*^, where Re is Reynolds number. Since the air flowing under the collector is turbulent, according to the literature (Zukauskas and Slanciauskas, 1987), we use the equation *Sh = 0,032 × Re* ^*0.8*^ *× Sc* ^*0.43*^ to calculate Sh.

Table 4 shows the velocity V_c_, Re, Sh, and the corresponding mass transfer coefficients at different positions under the collector of a 50 kW SUT system, which can be used for qualitative analysis of mass transfer under collector. It can be seen that at the entrance to the collector, the air velocity is the lowest and the corresponding mass transfer rate constant is the smallest. As the air flows to the center of the collector, the velocity increases and mass transfer coefficient also increases. It is because the higher velocity enhances the air turbulence, which helps to enhance the mass transfer of methane in the air, so the mass transfer coefficient increases. The mass transfer coefficient ranges from 0.0019 to 0.0191 m/s, which is in accordance with the previous results calculated by Reynolds analogy. The mass transfer rates at different positions of the collector can be estimated according to Eq. 3 and list in Table 4.

**TABLE 4:** Key parameters at different positions under the collector for 50 kW SUT.

Radius	V_c_, m/s	Re	Sh	h_m_, m/s	W(CH_4_), mol m^−2^ s^−1^
122 (From the entrance to the collector)	0.51	116,944	313	0.0019	1.32 × 10^–7^
90	0.70	158,524	399	0.0025	1.68 × 10^–7^
60	1.05	237,786	552	0.0034	2.33 × 10^–7^
30	2.09	475,572	962	0.0060	4.05 × 10^–7^
0 (Bottom of the chimney)	9.00	2,046,369	3,091	0.0191	1.30 × 10^–6^

In the same way, Re, Sh, h_m_, W(CH_4_) of 200 MW SUT can be calculated easily, and listed in Table 5. Compared with 50 kW SUT, 200 MW SUT has higher air velocity and mass transfer rate.

**TABLE 5:** Key parameters for 200 MW SUT.

Location	V_c_, m/s	Re^a^	Sh	h_m_, m/s	W(CH_4_), mol m^−2^ s^−1^
The entrance to the collector	3.0	1,106,145	1890	0.0072	4.90 × 10^–7^
The bottom of the chimney	15	5,530,726	6,848	0.0261	1.78 × 10^–6^
Average velocity	5.8	2,149,609	3,215	0.0123	8.35 × 10^–7^

a The height of the collector is 3 m.

To evaluate the mass transfer process comprehensively, it is essential to calculate the average mass transfer coefficient. Because there is not enough data of heat transfer to obtain the average mass transfer rate coefficient by Reynolds analogy method, the average mass transfer rate coefficient is obtained by calculating the average flow rate. The velocity of the air under the collector can be written as following equation:

$$\frac{\text{d}R}{\text{d}t}=\frac{G}{2\pi RH_{c}},$$ (8)

where *t* is the residence time, min; G is the volumetric flow rate at the outlet of the tower, m^3^/s; ${\text{H}}_{\text{c}}\text{ is the average canopy height}.\;$

From Eq. 8, it can be estimated that the residence time of air under the collector is 10 min for 200 MW SUT. The average velocity is obtained by dividing the collector radius by the residence time. The mass transfer rate coefficient obtained from the average velocity is shown in Table 5.

### Analysis of Rate Limiting Step

By comparing the mass transfer rate W(CH_4_) with the reaction rate r(CH_4_), we can determine the rate-limiting step. 1) if W(CH_4_) >> r(CH_4_), reaction is the bottleneck of the whole process; 2) if W(CH_4_) << r(CH_4_), mass transfer is rate-limiting step, 3) if W(CH_4_) and r(CH_4_) are very close, it means that the reaction and mass transfer have equivalent influence and jointly control the whole process.

According to photocatalytic data from TiO_2_, the photocatalytic decomposition rate of methane is 4.8 × 10^–9^ mol m^−2^ s^−1^. No matter for 50 kW SUT or 200 MW SUT, even the minimum W(CH_4_) at the collector entrance is much greater than r(CH_4_), so photocatalytic reaction is the rate-limiting step.

The reaction rate based on 0.1-Ag-ZnO is 2.16 × 10^–7^ mol m^−2^ s^−1^, which is close to the diffusion rate. Therefore, for 50 kW SUT, catalytic reaction is the rate-limiting step in the area near the collector inlet, while diffusion is the rate-limiting step in the centre of the collector. For 200 MW SUT, photocatalytic reaction is the rate-limiting step under the whole collector.

### Estimation of $\mathrm{NT}U_{m}$ and ε

According to Eq. 1, the apparent reaction rate constant K_app_ can be estimated by the following equation for Haeger et al.’s model (Haeger et al., 2004a).

$$K_{app}=IB\frac{B_{1}}{1+B_{1}C\left(CH_{4}\right)}\cdot \frac{B_{2}C\left(O_{2}\right)}{1+B_{2}C\left(O_{2}\right)}.$$ (9)

The mass conservation equation together with the boundary conditions can be written as

$$-G\frac{\text{d}C\left(CH_{4}\right)}{2\pi R\;\text{d}R}=K_{app}C_{s}\left(CH_{4}\right)$$ (10)

$$K_{app}C_{s}\left(CH_{4}\right)=h_{m}\left(C\left(CH_{4}\right)-C_{s}\left(CH_{4}\right)\right)$$ (11)

$$\mathrm{R=}R_{\mathrm{coll}}\mathrm{, C}\left(CH_{4}\right)=C_{\mathrm{in}}\left(CH_{4}\right),$$ (12)

where *R*_*coll*_ is the radius of the collector; *C*_*in*_(CH_4_) is the inlet methane concentration; *K*_*app*_ is the rate constant of apparent reaction, m/s.

The solution to Eq. 10 is

$$C_{s}\left(CH_{4}\right)=\frac{1}{1+K_{app}/h_{m}}C\left(CH_{4}\right)$$ (13)

$$C_{out}\left(CH_{4}\right)=C_{in}\left(CH_{4}\right)e^{-K_{app}A_{r}/\left(\left(1+K_{app}/h_{m}\right)G\right)},$$ (14)

where *A*_*r*_ is the surface area of reaction, m^2^. C_out_(CH_4_) is the methane concentration in the outlet air. The methane removal effectiveness of a SUT can be characterized by the fractional conversion.

$$\text{ε}=\frac{C_{in}\left(CH_{4}\right)-C_{out}\left(CH_{4}\right)}{C_{in}\left(CH_{4}\right)},$$ (15)

If methane is completely decomposed, ε is 1. If no methane is decomposed, ε is 0.

The number of mass transfer units (NTU_m_) is defined as,

$$NTU_{m}=\frac{A_{r}}{G\left(1/K_{app}+1/h_{m}\right)},$$ (16)

Equation 16 is often used to assess the number of mass transfer units in a process that contains both physical mass transfer and chemical reaction.ε can then be written as

$$\varepsilon =1-e^{-NTU_{m}}.$$ (17)

The calculation results of NTU_m_ and *ε* are shown in Table 6. If the photocatalytic technology is applied to 50 kW SUT, the methane removal rates are 0.44 and 17.5% with TiO_2_ and 0.1-Ag-ZnO respectively, which are lower than the results previously analysed (de Richter et al., 2017) and the methane removal rates calculated in Table 2. The main reason is that the previously analysed results only consider the mass transfer process, while the removal rate in Table 2 only considers the photocatalytic reaction kinetics. But in fact, the whole process involves both photocatalytic reaction and mass transfer process. The methane removal rates in a 200 MW SUT are 1.4 and 42.5% with TiO_2_ and 0.1-Ag-ZnO respectively.

**TABLE 6:** NTU_m_ and ε of 50 kW and 200 MW SUT.TABLE 6NTU_m_ and ε of 50 kW and 200 MW SUT.

	TiO_2_		0.1-Ag-ZnO	
	50 kW	200 MW	50 kW	200 MW
$NTU_{m}$	4.5 × 10^–3^	1.4 × 10^–2^	0.193	0.554
ε	0.0044	0.014	0.175	0.425

## Further Discussion

### Photocatalysts

With the understanding of relevant mass transfer and kinetic phenomena, it is found that the overall rate of methane oxidation is limited by the adsorption and oxidation step rather than the diffusion of the species to the surface in most scenarios. Therefore, the future efforts should focus on improvement of photocatalytic materials.

**FIGURE 5:**
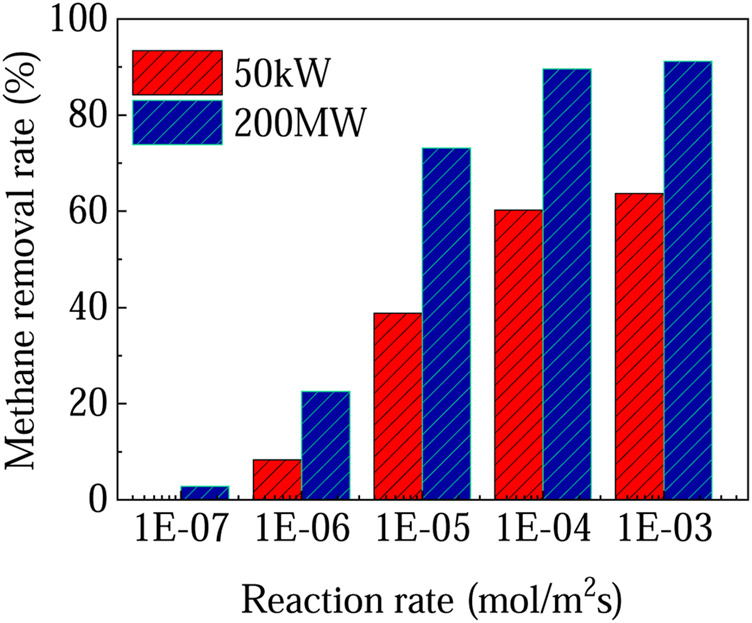
Methane removal rate at different reaction rates.

The above analysis is based on two typical photocatalysts for methane oxidation, i.e. less effective TiO_2_ and more effective 0.1-Ag-ZnO sample. It is found that the process is almost unfeasible due to the relatively low methane removal rate on TiO_2_. However, for 200 MW SUT, the methane removal rate can reach 42.5% if 0.1-Ag-ZnO is used. Figure 5 shows the methane removal rates can be further enhanced if even higher reaction rates can be achieved by developing even more advanced photocatalysts. For example, if the reaction rate reaches 1 × 10^–5^ mol m^−2^ s^−1^, the methane removal rate of 200 MW SUT can reach 73%. The development of photocatalysts with high reaction rate is undoubtedly the primary task.

### Catalyst Loading Amount (i.e. Coating Area)

**FIGURE 6:**
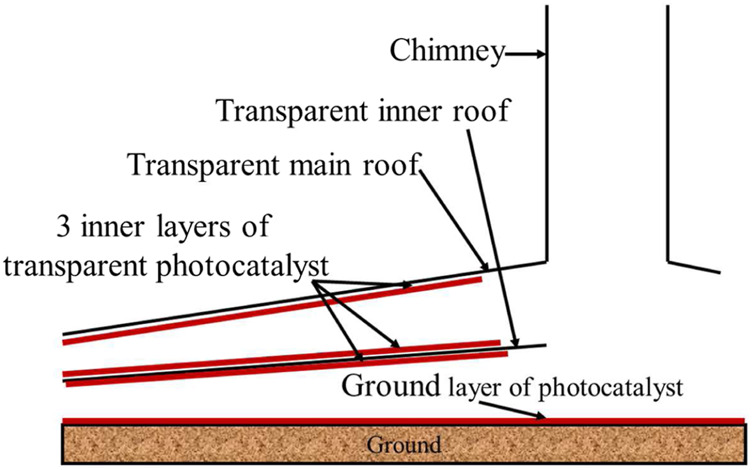
A SUT with double roof.

In addition to types of photocatalysts, we can also take some other strategies to improve the photocatalytic reaction rate, such as increasing the loading amount (i.e. reaction area). There are different ways to apply photocatalysts, e.g. on the surface under the canopy, or on the ground or both. Multi-layer roof is another method that can effectively increase the reaction area (Pretorius, 2007; de Richter et al., 2016), as shown in Figure 6. It should be pointed out that although the multi-layer roofs can effectively increase the reaction area, if too many layers are needed for sufficient contact area, perhaps poorer mass transfer would occur due to slower air flow and thicker boundary layers. The intensity of sunlight will also be weakened.

If the reaction rate of the photocatalyst is large enough to meet the reaction requirement only by coating part of the collector, it is suggested that the photocatalyst should be positioned toward the outer rim of the collector rather than the center. Under the collector, with the air flow to the center, the velocity steadily rises. The rise of velocity intensifies the air turbulence, which is beneficial to the mass transfer process. But it also reduces the contact time between methane and photocatalyst. Since the reaction is the bottleneck of the whole process, it is reasonable to coat the catalyst on the periphery of the collector.

### Light Intensity and Quantum Efficiency

Light availability and penetration to the catalyst could present a further limitation, according to the quantum efficiency of the photocatalytic reaction. During photocatalytic process where photons strike photocatalyst surface, stimulate charge carriers inside photocatalyst and then the charge carriers move to photocatalyst surface and react with CH_4_ molecules. Apparent quantum yield (AQY), defined in Eq. 18, is widely used in heterogeneous photocatalysis (solid/liquid or solid/gas systems) to quantify the efficiency of photocatalysts or photocatalytic processes.

$$\text{AQY}=\frac{\text{the number of reacted electrons}}{\text{the number of incident photons}}.$$ (18)

Assuming that only UV light can be used for the total oxidation of methane, and the ground under collector (46,760 m^2^) are equipped with photocatalysts, all methane passing through 50 kW SUT can be removed with an AQY of 5.2%. Similarly, we calculated the required AQY of 1.7% for a 200 MW SUT which has a greenhouse area of 3.8 × 107 m^2^, summarized in Table 7.

**TABLE 7:** Estimation of AQY required in a SUT for total removal of atmospheric CH_4_.

SUT	Light intensity of UV^a^, W m^−2^	Photon flux^b^, m^−2^ S^−1^	Illuminated area, m^2^	CH_4_ need to be oxidised^c^, S^−1^	Required AQY (%)
50 kW	56	9.9 × 10^19^	46,760	3.0 × 10^22^	5.2
200 MW	56	9.9 × 10^19^	3.8 × 10^7^	8.2 × 10^24^	1.7

a Sunlight at the zenith (1120 W/m^2^), proportions of the two wavelength ranges are estimated according to solar spectrum at sea level.

b 350 nm was used in the calculation.

c Atmospheric concentration of CH_4_ is taken as 1.8 ppm and eight electrons are needed to totally oxide one CH_4_ molecule.

### Night Operation Strategies

Another challenge of solar photocatalysis is night operation. On the ground under canopy, a thermal energy storage layer can be applied to store heat during the day and to release heat at night, driving airflow and allowing electricity production 24 h/day with no-intermittency (Ming et al., 2021). As a consequence, there can be some feasible strategy to utilize a small part of generated power to drive photocatalysis at night. Among those possibilities, at least two options for photocatalytic research are directly related.

1) Using artificial illumination at night onto all/part of the existing photocatalytic areas. In this option, almost all research outcomes about photocatalyst will stand and be applicable.
2) Considering the nature of methane at climatically relevant scale (i.e. significant amount of airflow and extreme dilution of the greenhouse gas), we can also propose another efficient and process intensified technology: an internal-illuminated monolithic photoreactor with distributed optical fibers. The technology is pioneered by Lin and Valsaraj in photocatalytic wastewater treatment (Lin and Valsaraj, 2005), adapted into a gas-liquid-solid multiphase photocatalysis by Du et al. (2008), and applied in gas phase photocatalysis by Liou (Liou et al., 2011) and Lu (Lu et al., 2016). Large surface area per unit volume as well as low pressure drop under quick flow rate provided by the monolith can be extremely beneficial. Side light optical fibres can distribute light into the entire inner surface of every channel.

### Dimension of SUT

1) Height of the tower

The tower height not only determines the energy output of SUT, but also affects the outlet velocity, which is related to the amount of air flowing through SUT, the velocity and the residence time of air under the collector.

It can be seen from Eq. 19 that the outlet air speed of the tower increases with the increase of tower height, which means that more air will flow through the SUT collector with high tower, and the contact time between methane and photocatalyst is shorter Therefore, on the premise of meeting the energy output requirements, lower tower is conducive to improve the methane removal rate.

$$v=\sqrt{2gH_{T}\frac{\Delta T}{Ta}}.$$ (19)

2) Radius of Collector

The large radius collector can provide larger contact area and longer residence time, but at the same time, it reduces the inlet velocity and thus weakens the mass transfer. For the process in which photocatalytic reaction is the rate-limiting step, it is suggested to build a collector with large radius. But larger collectors mean higher investment and maintenance costs. Therefore, the collector radius needs to be considered comprehensively. It should be pointed out that from the perspective of energy output, there is an up-limit on the radius of the collector, larger radius than that will not produce more power output (Li et al., 2012).

### Temperature

In respect to the reaction rate, although methane oxidation is an exothermal reaction, Chen et al. conducted experiments under different temperatures and concluded that temperature fluctuation has little effect on the photo-oxidation process (Chen et al., 2016). At the mass transfer side, status of air flow is the major factor for mass transfer. Air flow under the collector mainly depends on temperature change (∆T) and tower height (H_T_) (Schlaich et al., 2005), and the influence of ambient temperature (T_a_) on ∆T and H_T_ is negligible. Therefore, it is believed that temperature might not be a major influencer for air flow and thus should not be a major influencer for mass transfer.

## Conclusion

In this article, feasibility of SUTs as photocatalytic reactors for removal of atmospheric methane was evaluated. We examined the role of catalysts and rate limiting steps in particular.

The main findings are, 1) The effectiveness of combining photocatalysis with SUTs highly depends on the efficacy of photocatalysts and the size of SUT. More effective 0.1-Ag-ZnO photocatalyst can remove17.5 and 42.5% of methane in 50 kW and 200 MW SUTs respectively, while less effective TiO_2_ can only remove 0.4 and 1.4%. 2) If the more effective 0.1-Ag-ZnO photocatalyst is applied, in a 50 kW SUT, reaction and mass transfer jointly control the whole process, while in a 200 MW SUT, only reaction is the rate limiting step.

Outlook for future work to further improve the methane removal rate includes, 1) developing more effective photocatalysts, 2) increase catalyst coating area, 3) optimizing SUT geometry and 4) exploring night operation strategies.

## Data Availability

The original contributions presented in the study are included in the article/Supplementary Material, further inquiries can be directed to the corresponding author.
